# Evaluation of 71 Coronary Artery Disease Risk Variants in a Multiethnic Cohort

**DOI:** 10.3389/fcvm.2018.00019

**Published:** 2018-03-14

**Authors:** Wangjing Ke, Kristin A. Rand, David V. Conti, Veronica W. Setiawan, Daniel O. Stram, Lynne Wilkens, Loic Le Marchand, Themistocles L. Assimes, Christopher A. Haiman

**Affiliations:** ^1^Department of Preventive Medicine, Keck School of Medicine of USC, Los Angeles, CA, United States; ^2^Ancestry, San Francisco, CA , United States; ^3^Epidemiology Program, University of Hawaii Cancer Center, Honolulu, HI, United States; ^4^Department of Medicine, Stanford University School of Medicine, Stanford, CA, United States; ^5^Cardiovascular Institute, Stanford University School of Medicine, Stanford, CA, United States

**Keywords:** coronary heart disease, genome wide association study (GWAS), multi-ethnic, African Americans, Latino American, Japanese Americans, *SORT1*

## Abstract

**Background:**

Coronary heart disease (CHD) is the most common cause of death worldwide. Previous studies have identified numerous common CHD susceptibility loci, with the vast majority identified in populations of European ancestry. How well these findings transfer to other racial/ethnic populations remains unclear.

**Methods and Results:**

We examined the generalizability of the associations with 71 known CHD loci in African American, Latino and Japanese men and women in the Multiethnic Cohort (6,035 cases and 11,251 controls). In the combined multiethnic sample, 78% of the loci demonstrated odds ratios that were directionally consistent with those previously reported (*p* = 2 × 10^−6^), with this fraction ranging from 59% in Japanese to 70% in Latinos. The number of nominally significant associations across all susceptibility regions ranged from only 1 in Japanese to 11 in African Americans with the most statistically significant association observed through locus fine-mapping noted for rs3832016 (OR = 1.16, *p* = 2.5×10^−5^) in the *SORT1* region on chromosome *1p13*. Lastly, we examined the cumulative predictive effect of CHD SNPs across populations with improved power by creating genetic risk scores (GRSs) that summarize an individual’s aggregated exposure to risk variants. We found the GRSs to be significantly associated with risk in African Americans (OR = 1.03 per allele; *p* = 4.1×10^−5^) and Latinos (OR = 1.03; *p* = 2.2 × 10^−8^), but not in Japanese (OR = 1.01; *p* = 0.11).

**Conclusions:**

While a sizable fraction of the known CHD loci appear to generalize in these populations, larger fine-mapping studies will be needed to localize the functional alleles and better define their contribution to CHD risk in these populations.

## Introduction

Coronary heart disease (CHD) is the most common, chronic, life-threatening illness in the United States, affecting more than 11 million people (1). A study with twins has estimated the genetic contribution to the variation in CHD mortality to be 0.57 and 0.38 in males and females, respectively (2). Genome-wide association studies (GWAS) have been conducted primarily in populations of European ancestry and have identified ~65 regions associated with CHD risk (3–11). Many of the CHD loci were identified in a large study of 22,233 case and 64,762 control of European ancestry in the CARDIoGRAMplusC4D consortium, which reported 46 genome-wide significant variants with odds ratios ranging from 1.01 to 2.08 and effect allele frequencies of 0.06–0.91 (9). More recently, 10 additional loci were reported from the same consortium in a genome-wide association study involving 61,289 cases and 126,310 controls subjects following imputation to the 1,000 Genomes Project reference panel (12). Genome-wide scans have also revealed 7 CHD risk loci in Asian populations (13–17). The known genetic risk variants for CHD are estimated to explain only 10–11% of the heritability of CHD (9, 12), suggesting that many additional genetic susceptibility loci remain to be discovered.

Several studies in Asian populations have reported successful replication of known CHD regions (17–20), with a reproducible disease association consistently noted with the **9*p21* region. A limited number studies have been performed to investigate risk associated with CHD variants in minority groups such as African Americans or Latinos (21–28). In 2011, a GWAS in African Americans found a SNP, rs1859023, located at *7q21* near the *PFTK1* gene to be significantly associated with CHD (22), however this finding has never been replicated in African Americans or any other racial/ethnic group. In a study of 8,090 African Americans (~700 CHD cases) that examined known CHD risk regions, only *﻿﻿9p21*﻿ was found to be associated with CHD (25). In a study of 8,201 African Americans (~550 CHD cases) (26), investigators found consistent direction of effects compared to studies of European ancestry for 23 of 44 (binomial *p* = 0.52) known loci with two nominally statistically significant (rs599839 at *1p13/SORT1* and rs579459 at *4p23/ABO*). Genetic studies of CHD in Latino populations have been extremely limited. In a Costa Rican study that examined only 14 CHD SNPs in 1,898 cases with MI and 2,096 controls, 7 variants at 3 regions (*SORT1*,* CXCL12*, and*9p21*) were found to be significantly associated with risk (29). Thus, additional studies are needed to understand the generalizability and relevance of the known CHD risk loci in populations of non-European ancestry.

In this context, the objective of this study was threefold. First, we wished to determine whether associations involving 71 known susceptibility variants of CHD from 65 independent regions generalize across African-American, Latino and Japanese men and women in the Multiethnic Cohort, a study that includes over 6,000 cases and 11,000 controls. Second, we evaluated common genetic variation across each susceptibility region in an attempt to identify variation that might better define the risk associations compared to the index variants in the multiethnic sample. Lastly, we constructed genetic risk scores (GRS) summarizing one’s degree of exposure to high risk alleles of CHD and evaluated to what degree this GRS contributes to population differences in CHD risk.

## Methods

### Study Population

The Multiethnic Cohort study (MEC) is a large prospective cohort study that was established between 1993 and 1996. The MEC includes primarily African Americans, Japanese American, Native Hawaiians, Latinos and European Americans living in Hawaii and California. Cohort members were recruited through Department of Motor Vehicle license files and supplemented by voter registration and Health Care Financing Administration (Medicare) files. Participating individuals were between 45 and 75 years of age, and completed a 26-page self-administered, detailed questionnaire at cohort entry (baseline data, 1993–1996). The questionnaire included basic demographic factors (including race/ethnicity and education), lifestyle factors (e.g., diet, medication use and smoking history), and chronic medical conditions. Follow-up questionnaires were also administered in years 1999 and 2003 which contained updates on participant’s CHD status and lifestyle factors.

Several nested case-control studies have been assembled in the MEC for GWAS of a number of cancer and non-cancer traits (30–32) including breast cancer, prostate cancer, and type-2 diabetes, mainly in populations of non-European ancestry. In the current study, we identified CHD cases and non-cases within these nested studies for the genetic analysis of CHD risk SNPs.

The MEC study obtained written informed consent from study participants for genetic analysis, approval from the Health Science Review Board (HSIRB) at the University of Southern California, and IRB certification permitting data sharing in accordance with the NIH Policy for Sharing of Data Obtained in NIH Supported or Conducted Genome-Wide Association Studies (GWAS). Genetic data for the MEC is available on dbGAP (phs000517.v3.p1, phs000851.v1.p1, phs000356.v2.p1, phs000306.v4.p1, phs000683.v1.p1)

## CHD Case/Control Definitions

CHD cases were identified through linkage of the MEC to the California Hospital Discharge Data (1990–2012) (CHDD) and the Centers for Medicare and Medicaid Services (CMS) claim files (MedPAR, outpatient) (1999–2011). Hospital discharge information was not available for the subjects from Hawaii which included 76.6% of the Japanese men and women. A CHD case was defined as having ischemic heart disease under ICD-9 codes (DX 410–414), by the principal or first diagnosis code and the principal or first procedure code. We also included cases with a primary cause of death due to myocardial infarction (ICD-9 DX410, ICD-10 I21), or other CHD conditions (ICD-9 DX411–414, ICD-10 I20, I22–25). Both prevalent and incident CHD cases were included in this study. Of the 6,035 CHD cases identified, 1,146 were identified from their baseline questionnaires at the time of enrollment in the MEC study, and a majority of these prevalent cases (1,122, 97.9%) were also identified from CHDD or Medicare.

Controls in this study were subjects with no history of heart attack or angina based on the baseline questionnaire or all subsequent follow-up questionnaires. Those taking nitrates at blood draw in subsequent examinations were also excluded. Individuals with non-primary CHD diagnosis codes (i.e., 2–24) from the CHDD and Medicare data were excluded from being either a case or control. A total of 11,251 controls were selected, of which 8,307 had at least one previous Medicare or CHDD claim (and thus would have been identified as a case). A sensitivity analysis using controls with definite claim information was performed.

## Genotyping and Quality Control

We utilized genetic data generated from case-control studies in the MEC of breast cancer, prostate cancer, and type 2 diabetes in African Americans (2,976 males and 3,539 females), Japanese Americans (2,530 males and 2,132 females), and Latinos (3,340 males and 2,769 females). Genotyping was conducted using the Illumina platform with different arrays, including the Human 1M-Duo v3.0 BeadChip (31, 32), HumanOmni2.5-Quad BeadChip (33), Human 660W-Quad BeadChip (34), and the Cardio-MetaboChip (35) (Table S1). We used the following exclusion criteria to remove samples whose genetic or phenotypic data were questionable: (1) unknown replicates across studies, (2) call rates < 95%, (3) samples with mismatched gender, such as male samples with >10% mean heterozygosity of SNPs on the X chromosome and/or <10% mean intensity of the Y chromosome; or female samples with <15% mean heterozygosity of SNPs on the X chromosome and/or similar mean allele intensities of SNPs on the X and Y chromosomes, (4) ancestry outliers (>4 standard deviations from the mean of the first or second principal component), and (5) first degree relatives.

A subset of 2,717 African Americans (879 CHD cases and 1,838 controls) and 1,184 Japanese Americans (302 CHD cases and 882 controls) genotyped with the Cardio-MetaboChip were missing data for 20 of the 71 SNPs; these subjects were excluded from the risk score analysis.

## SNP Imputation and Principal Components Analysis

All samples except for the African-American and Japanese samples genotyped with the Metabochip were imputed using the software IMPUTE2, based on build 37 (hg19) coordinates, to the 1000 Genome Project data phase 1 v3. Principal components were calculated by study in smartpca from EIGENSOFT (36) using a random selection of 10,000 SNPs across the genome (MAF >5% and call rate >95%).

## Statistical Analysis

The log-additive effect of each SNP on CHD risk was estimated in PLINK using unconditional logistic regression adjusted for age, sex, BMI and the first 10 principal components to account for potential population stratification (37). All analyses were stratified by ethnicity, disease status (i.e., breast cancer, prostate cancer, or type 2 diabetes disease status). METAL was used to combine the results within and across populations, which included 18 case-control strata in the overall meta-analysis of all populations. For SNPs that were imputed, all were imputed with an IMPUTE2 INFO score >0.8 in each study and population. SNPs rs11752643 and rs3782886 in African Americans, rs180803 in Latinos, and rs6544713, rs4252120, rs2023938, rs3918226, rs3184504, and rs9982601 in Japanese had a minor allele frequency less than 1% and were not included in the ethnic-specific analysis. The cross-ethnic meta-analysis was performed on SNPs observed in at least two ethnic groups.

In addition to testing of the index SNP, we examined regional replication of the signal through testing SNPs in linkage disequilibrium (LD) with the index SNP in European ancestry groups (r^2^ ≥0.4 in EUR 1000 Genomes Project). Haploview (38) was used to assess pairwise tag SNPs among bins of markers in the AFR population [tagging r^2^ ≥0.8 for SNPs with a MAF >1% based on 1000 Genomes Project data (39)]. For each region, an alpha threshold of significance was set at 0.05 divided by the number of tag SNPs in AFR. We considered evidence of replication to be present in a region when one or more SNPs in LD with the index SNP had a *p*-value that was lower than the region-defined alpha threshold. For imputed SNPs, only those imputed with high quality (IMPUTE2 INFO score >0.8) were included in the regional replication testing. The regional association plots were generated with the LocusZoom program (40).

We also examined the aggregate effect of the CHD risk loci. Three genetic risk scores (GRS) were calculated for each individual: (1) An unweighted GRS comprised of risk-associated alleles from the 71 CHD SNPs, (2) a modified unweighted GRS (I) that substitutes the index SNP with the lead SNP reaching region-wide significance within a specific race/ethnic group of each known CHD locus, and (3) a modified unweighted GRS (II) similar to I but substituting index SNPs with the leading SNPs in each region from our cross-ethnic meta-analysis. The risk alleles for the substitution SNPs were determined based on their observed effects in our study. As outlined above, subjects genotyped with the non-GWAS Metabochip were excluded from the risk score analysis because of missing data on 20 SNPs. The risk score distributions across ethnic groups were compared using a two-sided *t*-test. The association of genetic risk scores with CHD were evaluated within each ethnicity in a logistic regression model adjusted for age, sex, BMI, and the first 10 principal components. Of the 71 SNPs selected, only one pair (rs16986953 and rs2123536) from *TTC32-WDR35* was correlated. Since the association between rs2123536 and CHD was only observed in a Chinese population (16), both SNPs were kept in the GRS analysis.

Within each population, statistical power for each SNP was calculated in the R package “gap,” (41) using the allele frequency in each racial/ethnic group, and the documented OR from the literature. The allele frequency for the multiethnic sample was weighted by the sample size of each ethnic group. The power for detecting rare and common alleles within each ethnic group was calculated using QUANTO (42).

## Results

Descriptive characteristics of the 6,035 CHD cases and 11,251 controls stratified by sex and race/ethnicity are presented in Table 1. We analyzed a total of 2,376 African-American cases and 4,139 controls, 2,291 Latino cases and 3,818 controls, and 1,368 Japanese cases and 2,294 controls. In general, compared to controls, CHD cases were slightly older at cohort entrance, were heavier in all three ethnic groups and were more likely to have ever smoked than controls in all three ethnic groups (Table 1). The associations of BMI and smoking with CHD were similar when further stratified by prevalent conditions, including prostate cancer, breast cancer, and diabetes (Table S2).

**Table 1:** Descriptive Characteristics of CHD Cases and Controls

**Study Population (Total *N* = 17286, CHD Case *N* = 6035, CHD Control *N* = 11251)**					
		**African Americans (*N* = 6515)**			
		**Male (*N* = 2976)**		**Female (*N* = 3539)**	
		**CHD cases (*N* = 1234)**	**CHD controls (*N* = 1742)**	**CHD cases (*N* = 1142)**	**CHD controls (*N* = 2397)**
Ever smoked^*^	Yes (%)	936 (75.85)	1205 (69.17)	654 (57.27)	1120 (46.73)
	No (%)	289 (23.42)	524 (30.08)	470 (41.15)	1257 (52.44)
BMI	Mean (SD)	27.60 (4.29)	27.42 (4.24)	30.06 (6.10)	28.71 (5.76)
Age at cohort entry	Mean (SD)	63.90 (7.22)	60.16 (8.64)	62.30 (8.05)	57.29 (8.71)
		**Latinos (*****N***** = 6109)**			
		**Male (*****N***** = 3340)**		**Female (*****N***** = 2769)**	
		**CHD cases (*****N***** = 1364)**	**CHD controls (*****N***** = 1976)**	**CHD cases (*****N***** = 927)**	**CHD controls (*****N***** = 1842)**
Ever Smoked^*^	Yes (%)	976 (71.56)	1306 (66.09)	336 (36.25)	599 (32.52)
	No (%)	357 (26.17)	638 (32.29)	554 (59.76)	1173 (63.68)
BMI	Mean (SD)	27.72 (4.01)	27.43 (3.78)	29.37 (5.96)	27.81 (5.19)
Age at cohort entry	Mean (SD)	62.67 (6.31)	59.62 (6.98)	61.25 (6.49)	57.71 (7.16)
		**Japanese Americans (***N*** = 4662)**			
		**Male (***N*** = 2530)**		**Female (***N*** = 2132)**	
		**CHD cases (*****N***** = 930)**	**CHD controls (*****N***** = 1600)**	**CHD cases (*****N***** = 438)**	**CHD controls (*****N***** = 1694)**
Ever Smoked^*^	Yes (%)	664 (71.40)	1050 (65.63)	138 (31.51)	515 (30.40)
	No (%)	262 (28.17)	541 (33.81)	296 (67.58)	1170 (69.07)
BMI	Mean (SD)	25.50 (3.18)	25.56 (3.53)	24.40 (4.31)	24.25 (4.11)
Age at cohort entry	Mean (SD)	64.30 (6.94)	59.71 (8.76)	62.26 (7.09)	57.20 (8.24)

*^a^*Numbers don’t total to 100% due to missing data.

We had *a priori* greater than 80% power to detect reported per allele effect sizes for 6 out of 71 SNPs in African Americans, 9 out of 71 SNPs in Latinos, and 9 out of 71 SNPs in Japanese Americans and 16 out of 71 SNPs when combining samples from all three ethnic groups (Figure S1). Given the sample size in each ethnic group, we had 28.5% power to detect an OR of 1.12 (mean OR from the selected index SNPs) for a rare (MAF = 0.05) allele and 71.6% power to detect the same OR for a common (MAF = 0.20) allele in African Americans; we had 27.3% power to detect OR of 1.12 for a rare allele and 69.3% power for a common allele in Latinos; and 20% power for a rare allele and 52.7% power for a common allele in Japanese Americans.

We examined evidence of replication for 71 CHD variants from 65 regions (Table S3). Among these variants, 69 in African Americans, 70 in Latinos, and 65 in Japanese Americans had a MAF >1% and were included in the analysis. Compared to the null expectation that one-half of the examined SNPs show consistent direction of effects as previously reported, 65.2% (45 of 69, binomial *p* = 0.008) SNPs in African Americans, 70.0% (49 of 70, binomial *p* = 5.5 × 10^−4^) in Latinos, 58.5% (38 of 65, binomial *p* = 0.11) in Japanese, and 77.5% (55 of 71, binomial *p* = 2.0 × 10^−6^) in the combined multiethnic sample had the same direction of association as previously reported. In African Americans, nominally statistically significant associations (*p* < 0.05) and consistent directional effects were observed for 11 index SNPs in *PPAP2B*, *SORT1*, *IL6R*, *REST-NOA1*, *BTN2A1*, *SLC22A3-LPAL2-LPA*, *﻿9p21*, *CXCL12*, *SH2B3*, and *KCNE2*. In Latinos, nominal evidence of association (*p* < 0.05) and consistent directional effects were observed with 8 index SNPs at *SORT1*, *APOB*, *NOS3*, *LPL*, *ZHF259-APOA5-APOA1*, *MFGE8-ABHD2*, *FURIN-FES*, and *BCAS3*. In Japanese, only 1 index SNP at *﻿9p21* was nominally significant and directionally consistent. In the combined multiethnic sample, 10 index SNPs at *PPAP2B*, *SORT1*, *IL6R*, *REST-NOA1*, *EDNRA*, *PHACTR1*, *BTN2A1*, *NOS3*, *﻿9p21*, and *CXCL12* were directionally consistent and nominally statistically significant.

We observed evidence of regional replication for 6 regions in African Americans, 3 in Latinos, 1 in Japanese Americans, and 10 in the combined sample when examining SNPs correlated with the index SNPs (Table S4; see Methods). The previously reported index SNP in four of the 10 regions was not significant at the 0.05 level, but correlated SNPs with p-values smaller than the region specific significance levels were detected in these four regions: *SLC22A4-SLC22A5* and *RAI1-PEMT-RASD1* in African Americans, *TTC32-WDR35* in the multiethnic analysis, and *APOE-APOC1* in Latinos and the multiethnic sample.

**Figure 1:**
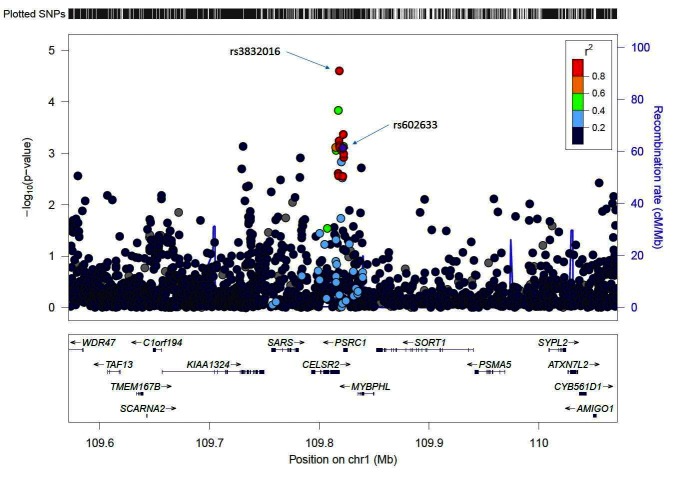
Results for the *SORT1* region on chromosome 1p13 from the multiethnic meta-analysis. The r^2^ shown is for the EUR group in the 1000 Genomes Project relative to the index SNP rs602633. The stronger signal, rs3832016, is also highlighted. This regional association plot was generated with the LocusZoom plot (40).

The most statistically significant association was observed at the *SORT1* locus (Figure 1). Two index SNPs in complete LD (rs602633 and rs599839) were initially reported from GWAS in European ancestry populations. The index SNP rs602633 was associated with risk in African Americans (OR = 1.13; *p* = 0.004), Latinos (OR = 1.11; *p* = 0.04), and in the cross-ethnic meta-analysis (OR = 1.11; *p* = 7.8×10^−4^), but not in Japanese Americans (OR = 1.01, *p* = 0.88). The most significant association in the region was with variant rs3832016 (OR = 1.16; *p* = 2.5×10^−5^ in the multiethnic sample), an INDEL (−/T) in high LD with rs602633 in EUR (r^2^ = 0.96) and with a MAF of 0.35 in African Americans, 0.20 in Latinos, and 0.07 in Japanese Americans. A previous fine-mapping study of the *SORT1* region at *﻿1p13* implicated a nearby non-coding polymorphism (rs12740374) to be the likely functional variant and to affect lipoprotein metabolism (43). SNP rs12740374 is in high LD not only with the index SNP rs602633 (r^2^ = 0.90 in EUR) but also with rs3832016 (r^2^ = 0.94 in EUR). Variant rs12740374 was less strongly associated with risk in the current study (*p* = 0.008 in African Americans with MAF = 0.25, *p* = 0.08 in Latinos with MAF = 0.20, *p* = 0.93 in Japanese Americans with MAF = 0.07, and *p* = 0.003 in the combined multiethnic analysis).

Other regions where evidence of regional replication was observed in African Americans include *PPAP2B* (rs72664341, *p* = 0.00018), *SLC22A4-SLC22A5* (rs17689550, *p* = 0.006), *SLC22A3-LPAL2-LPA* (rs4709431, *p* = 0.0077), *SH2B3* (rs10774625, *p* = 0.0047) and *RAI1-PEMT-RASD1* (rs9899364, *p* = 4.5 × 10^−4^). In Latinos, evidence for regional replication was observed at *MFGE8-ABHD2* (rs8037001, *p* = 0.0017), *FURIN-FES* (rs8182016, *p* = 1.1 × 10^−4^), and *APOE-APOC1* (rs7412, *p* = 0.0043). In the Japanese, regional replication was only observed at *﻿9p21* (rs10811656, *p* = 0.0015). Five of the 10 regions that replicated in the multiethnic analysis were also significant in ethnic-specific analyses, whereas the remaining 5 regions were detected with significant regional associations in one or more of the ethnic-specific populations alone (*TTC32-WDR35, APOB, EDNRA, PHACTR1* and *BCAS3*; Table S4).

**Figure 2:**
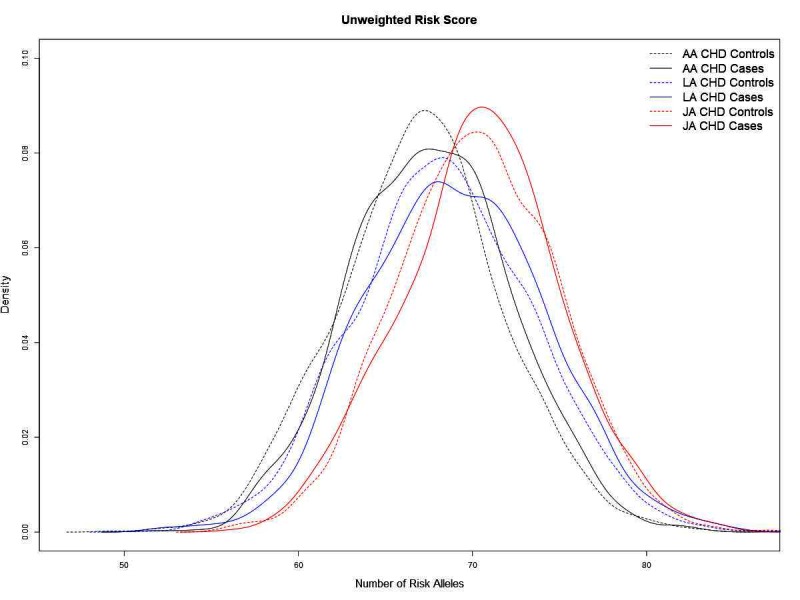
A comparison of the aggregate allele count risk score for cases and controls in each race/ethnic group.

Genetic risk scores (GRSs) were used to compare the distribution of genetic risk between populations. Japanese Americans carried, on average, more risk alleles (70.26 ± 4.61, mean ± SD) in comparison to African Americans and Latinos (67.03 ± 4.73 and 68.37 ± 5.11, respectively) (Table 2; Table S5). The greater number of risk alleles resulted in the distribution of the GRS to be shifted to the right in Japanese Americans compared to African Americans and Latinos (Figure 2). The distribution of the GRS was slightly higher in cases than in controls for every group (two-sided t-test, AA *p* = 4.4 × 10^−5^, LA *p* = 4.5 × 10^−7^, and JA *p* = 0.28). Only minor changes in the distribution of the GRS were noted when we included regionally significant leading SNPs from each ancestry (modified risk score I), or from the cross-ethnic meta-analysis (modified risk score II) (Table S5). The average risk scores remained highest in Japanese Americans whereas differences between African Americans and Latinos were reduced, especially when comparing CHD cases from these two ethnic groups (modified risk score II, *p* = 0.14).

**Table 2:** Associations of the genetic risk score with CHD by ethnicity.

		African Americans		Latinos		Japanese Americans	
CHD Risk Score(Mean ± SD, *p*)		Case = 67.67 ± 4.63Control = 67.03 ± 4.73p^*^ = 4.4 × 10^−5^		Case = 69.05 ± 5.13Control = 68.37 ± 5.11p^*^ = 4.5 × 10^−7^		Case = 70.44 ± 4.60Control = 70.26 ± 4.61p^*^ = 0.28	
Unweighted risk score		**OR**^***†***^	***p***^***†***^	**OR**^***b***^	***p***^***†***^	**OR**^***†***^	***p***^***†***^
	Continuous	1.031	4.09E-5	1.031	2.23E-8	1.014	0.11
	Quartile 1	Reference		Reference		Reference	
	Quartile 2	1.084	0.42	1.048	0.55	1.001	0.99
	Quartile 3	1.132	0.21	1.190	0.03	1.288	0.02
	Quartile 4	1.397	6.39E-4	1.393	1.98E-5	1.093	0.42
Modified risk score I^‡^	Continuous	1.028	2.12E-4	1.022	5.27E-5	1.009	0.29
	Quartile 1	Reference		Reference		Reference	
	Quartile 2	1.033	0.75	1.085	0.30	1.107	0.36
	Quartile 3	1.252	0.02	1.205	0.02	1.214	0.08
	Quartile 4	1.285	0.01	1.263	0.003	1.081	0.48
Modified risk score II^*§*^	Continuous	1.018	1.71E-3	1.028	9.06E-8	1.016	0.045
	Quartile 1	Reference		Reference		Reference	
	Quartile 2	0.976	0.81	1.066	0.42	1.062	0.59
	Quartile 3	1.278	0.01	1.182	0.03	1.342	0.007
	Quartile 4	1.280	0.01	1.393	2.0E-5	1.175	0.15

^*^Two-sided t-test

^*†*^Logistic regression model adjusted for age, gender, BMI, and the first 10 principal components

^*‡*^Risk score that includes ethnic-specific regional leading SNPs

^*§*^Risk score that includes cross-ethnic regional leading SNPs

The unweighted risk scores were statistically significantly associated with CHD risk in African Americans (per allele OR = 1.03, *p* = 4.1 × 10^−5^) and Latinos OR = 1.03, (*p* = 2.2 × 10^−8^), but only weakly associated with CHD risk in Japanese Americans (OR = 1.01, *p* = 0.11) (Table 2). When comparing individuals within GRSs in the top quartile to individuals in the bottom quartile, we found both African Americans (OR = 1.40) and Latinos (OR = 1.39) to have a statistically significant ~40% increase in risk (Table 2). The analogous risk was lower (~10%) and not significant in Japanese-Americans (OR = 1.09). Results were similar for the modified risk scores (Table 2).

To evaluate the effect of existing conditions on the results, we repeated the analysis excluding cancer or diabetes cases; the ORs were comparable to those observed in each ethnic group and in the entire sample (Table S6).

A sensitivity analysis was also performed on the selected index SNPs using controls refined to those with medical claims from Medicare or CHDD. Despite the loss of statistical power due to smaller sample size, the effect sizes were comparable to those observed when using the entire control sample (Table S7).

## Discussion

We evaluated 71 SNPs associated with CHD risk within 65 risk regions in a large multi-ethnic sample of African Americans, Latinos, and Japanese Americans and found that a statistically significant proportion of SNPs exhibited consistent directions of effect beyond the 50% expected by chance. However, only a subset of 11, 8, and 1 of these SNPs were found to be nominally statistically significant in African Americans, Latinos, and Japanese Americans, respectively. Exploration of common genetic variation in these CHD-associated regions provided additional support for association at 10 regions, with different ethnic-specific or cross-ethnic leading SNPs. These replication results provide additional evidence for shared common genetic effects across ethnicities, with previous studies only replicating signals at *9p21* (21, 24–26), *SORT1* (26), and *ABO* (26) in African Americans, and *SORT1*,* CXCL12*, and*9p21* in Latinos (29). This is the first report of *BTN2A1*, a region initially reported in Japanese, replicating in African Americans.

Japanese Americans had a higher GRS on average when compared to African Americans and Latinos. However, the GRS was more strongly associated with CHD in African Americans and Latinos compared to the Japanese Americans. We note that the genetic markers reported from previous discovery efforts are unlikely to be the functional alleles. The correlation between the index and functional SNPs may vary depending on the LD structure of each ancestral group, which may contribute to the difference in the ethnic-specific odds ratios. In addition to having limited statistical power to replicate associations with index SNPs within and across these populations, differences in LD may serve as an alternative explanation for the lack of replication. In an attempt to address such issues, we conducted regional association testing and constructed modified risk scores incorporating regional association results. When substituting the index SNPs with leading SNPs from the regional analyses, the differences in the modified risk score distributions and per-allele aggregate effects were only modified slightly, but differences were still noted, particularly between the Japanese and the other populations. The reasons for these differences are unclear. Our findings may reflect the severity of subclinical coronary atherosclerosis among Japanese participants in the MEC that is on average greater than the severity observed in Africans and Hispanics (44). Although our analyses are preliminary, we deem it unlikely that these known risk alleles are major contributors to race/ethnic differences in the incidence of CHD, as the incidence of CHD in Japanese is lower than that in the other two groups (45). Given that Japanese had a higher average GRS compared to other ethnic groups, but their population risk is lower, it is possible that functional variants within CHD susceptibility genes not included in our GRS disproportionally affect non-Japanese race/ethnic groups. Alternatively, environmental risk factors such as suboptimal diet and smoking may be less prevalent in Japanese and primarily responsible for the lower rates of CHD despite the higher genetic risk. It is difficult to directly compare the GRS distribution reported in this study to those in studies in European ancestry populations as the methods and number of selected SNPs vary (46–53). The vast majority of studies in European ancestry populations have observed statistically significant per allele relative risks of 1.02–1.12 and relative risks of 1.5–1.9 in comparing the highest versus lowest quintile or quartile of the GRS. Our findings in African Americans and Latinos are generally consistent with these reports albeit smaller effect sizes were noted, perhaps due to differences in LD between the index and functional SNPs.

Our study has a number of limitations. First, the information used to define CHD cases and controls was based on a combination of health care claims data as well as self-report on questionnaires. Some of the Japanese cases from Hawaii may have been missed due to the lack of CHDD records. Of the 1,089 Japanese participants whose CHDD records were available (in California), 426 CHD cases were identified, with 103 classified as cases based solely on CHDD records. Given the same ratio, about 338 Japanese CHD cases from Hawaii where CHDD was not available, may have been misclassified as controls. Assuming an equal distribution of genotypes in these missed cases compared to recognized cases, this misclassification would result in effects being biased towards the null and a reduced power to detect associations. Similar misclassification may apply as Medicare or CHDD data were not available for all controls. In the sensitivity analysis, limiting controls to those with claims data, fewer SNPs reached nominal statistical significance (0.05) however effect sizes were relatively comparable to those observed in the entire control sample. In an attempt to increase specificity when using Medicare and CHDD claims, we only included CHD cases identified from the primary and the first diagnosis codes, with individuals identified with CHD beyond the primary diagnosis excluded from being a case or a control. The validity of our case and control definitions is indirectly supported by the observed associations of case-control status with known risk factors and by the detection of more directionally consistent genetic associations than expected. Another limitation is the potential inclusion of inappropriately labeled CHD deaths, as CHD is often reported on death certificates when the cause of death is unclear. However, of the 1,005 CHD deaths, 718 also had prior claims data from CHDD or Medicare (Figure S2), suggesting a high consistency between mortality records and health care claims.

Another limitation of the study was the selection of CHD cases and controls among MEC participants conditional on three existing medical conditions. However, in sensitivity analyses limited to those without existing conditions, we observed robust consistency in terms of effect size and effect direction between this subset and the entire sample.

We present the largest replication study of established GWAS loci for CHD in Latinos and Japanese Americans conducted to date. However, our power was still limited to detect the originally reported effect sizes even in the combined multiethnic sample. We observed a higher GRS in Japanese Americans compared with African Americans or Latinos but the GRS was paradoxically not significantly associated with CHD risk in Japanese Americans despite observing strong associations in the other two groups. Substantially larger samples that include multiple racial/ethnic groups will help to identify the functional alleles in these regions and characterize their associations with CHD risk and contributions to CHD disparities among ethnically diverse populations.

## Ethics Statement

The MEC study obtained informed consent from study participants and approval from the Health Science Review Board (HSIRB) at the University of Southern California and obtained IRB certification permitting data sharing in accordance with the NIH Policy for Sharing of Data Obtained in NIH Supported or Conducted Genome-Wide Association studies (GWAS).

## Author Contributions

WK conducted the analysis and wrote the paper. KR contributed to the analysis. DC contributed to the analysis. VS contributed data for the manuscript. DS contributed to the analysis of the manuscript. LW contributed data for the manuscript. LL contributed data for the manuscript. TA contributed data and assisted with the writing of the manuscript. CH oversaw the project, contributed to the data, analysis and writing of the manuscript.

## Conflict of Interest Statement

Author KR is currently employed by company Ancestry.com. All other authors declare no competing interests.

The reviewer IB and handling Editor declared their shared affiliation.

## Supplementary material

Table S1 Genetic arrays by studies.

Table S2 Detailed demographic information on participants by ethnicity, gender, and existing conditions.

Table S3 Associations with CHD SNPs in the MEC populations.

Table S4 Results of regional replication by index or leading SNPs.

Table S5 Comparison of risk score distribution.

Table S6 Sensitivity analysis: comparing subpopulation with/without existing conditions.

Table S7 Sensitivity analysis: associations with CHD SNPs using controls with claims.

Figure S1 Statistical power for known CHD SNPs.

Figure S2 CHD case sources venn diagram.
