# Supplementary figures and images for: Evaluation of 71 Coronary Artery Disease Risk Variants in a Multiethnic Cohort
Source: Front Cardiovasc Med. 2018 Mar 14;5:19. doi: 10.3389/fcvm.2018.00019 (PMC5931137; doi:10.3389/fcvm.2018.00019)

Supplementary Figure 1. Statistical Power for Known CHD SNPs (some dots overlaps with each other)

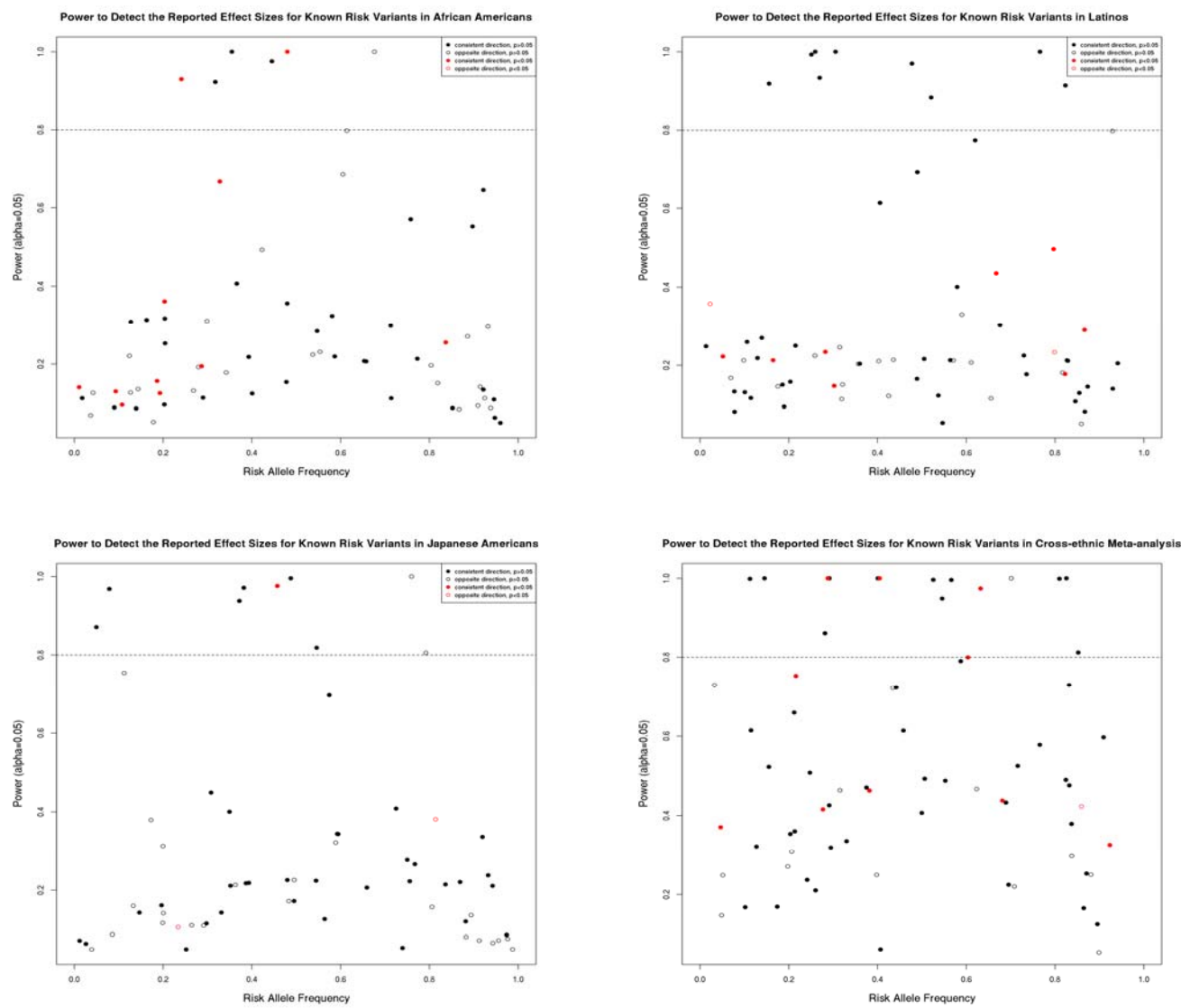

Supplement: Figure S1 — Statistical power for known CHD SNPs. [file Image1.pdf]

Supplementary Figure 2. CHD Case Sources Venn Diagram

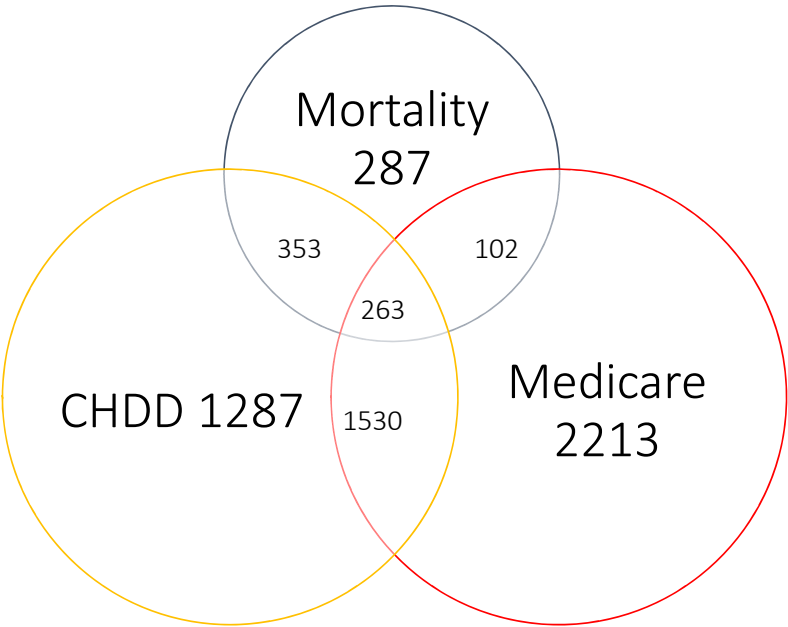

Supplement: Figure S2 — CHD case sources venn diagram. [file Image2.pdf]
